# The PI3Kδ inhibitor idelalisib impairs the function of human dendritic cells

**DOI:** 10.1007/s00262-021-02988-3

**Published:** 2021-06-25

**Authors:** Christiane Braun, Sebastian Schlaweck, Solveig Nora Daecke, Peter Brossart, Annkristin Heine

**Affiliations:** 1 Medical Clinic III, Clinic for Oncology, Hematology, Immuno-Oncology and Rheumatology/Clinical Immunology, University Hospital Bonn, Venusberg Campus 1, 53127 Bonn, Germany; 2 Faculty of Medicine, Mildred Scheel School of Oncology Aachen Bonn Cologne Düsseldorf (MSSO ABCD), University Hospital of Bonn, 53127 Bonn, Germany

**Keywords:** Idelalisib, Infection, Dendritic cells, PI3Kδ

## Abstract

The PI3Kδ-inhibitor Idelalisib is approved for the treatment of Non-Hodgkin lymphoma. However, its use has been decreased within the last years due to deleterious infections such as cytomegalovirus and pneumocystis jirovecii. Here, we have investigated the effect of Idelalisib on human monocyte-derived dendritic cells (DCs) as important players in the induction of immune responses. We found that Idelalisib-treated DCs displayed impaired T cell stimulatory function. PI3Kδ inhibition during differentiation resulted in decreased Interleukin-12, Interleukin-13 and TNFα production by DCs after lipopolysaccharide stimulation. Moreover, DCs showed decreased expression of the activation marker CD83 after Idelalisib treatment. Further, in line with this was the failure of Idelalisib-treated DCs to properly induce allogeneic T cells in a dose-dependent manner. Finally, activation of the NFκB pathway was also ablated in Idelalisib-treated DCs. Our results implicate that severe infectious complications may not only result from direct PI3Kδ-inhibition in T cells, but also from impaired DC function in Idelalisib-treated patients. Here, we provide new insight into the pathogenesis of Idelalisib-associated infectious complications. Our study may further provide a rationale for the use of Idelalisib as a novel therapeutic option in inflammatory diseases.

## Introduction

Non-Hodgkin lymphoma, like follicular lymphoma (FL) and small lymphocytic lymphoma/chronic lymphatic leukemia (SLL/CLL), are hematological malignancies characterized by uncontrolled proliferation of clonal B cells, which leads to painless lymphadenopathy in most cases. Any organ may be affected, which is reflected by the broad variety of clinical presentations of these diseases [1]. Treatment options for Non-Hodgkin lymphomas have evolved in the past decades from classical chemotherapy to immunotherapy as well as targeted therapy. Among targeted therapies, PI3Kδ has been identified as a potent target for B cell malignancies. PI3Kδ is a tyrosine kinase predominantly expressed in leukocytes. It is located in the cytoplasm and activates the AKT/mTOR pathway [2]. Idelalisib, a specific small molecule inhibiting PI3Kδ, showed remarkable clinical results for the treatment of FL as well as CLL [3, 4]. However, infection rates, for example of cytomegalovirus (CMV) and pneumocystis jirocevii (PJP) in patients treated with Idelalisib in combination with classical immune-chemotherapy, were significantly increased [5]. Impaired T cell-mediated immunity due to direct effects on T cell migration and cytokine production has already been described as a side effect of Idelalisib treatment [6].

T cell-mediated immunity and the induction of a potent immune response are not only T cell-dependent, but also require professional antigen-presenting cells (APCs). Dendritic cells (DCs) presenting a specific antigen and expressing appropriate co-stimulatory molecules are the most potent APCs. They can orchestrate immune responses of naïve CD8+ T cells and subsequently induce cytotoxic T cell responses [7].

In this study, we investigated the effect of Idelalisib on DC function, which is a prerequisite for appropriate T cell activation, differentiation and proliferation. Our study may highlight how PI3Kδ inhibition not only diminishes T cell function directly, but also affects antigen-specific T cell responses through attenuated DC function.

## Methods

### Samples

Human monocytes were isolated from buffy coats from voluntary blood donors at the University Hospital Bonn.

### Media and reagents

Idelalisib was purchased from Selleckchem. Cells were cultured in RPMI 1640 containing glutamax-I, supplemented with 10% inactivated fetal calf serum (RP10 medium) and 1% penicillin/streptomycin (Invitrogen). Unless otherwise indicated, all reagents were purchased from Sigma-Aldrich.

### Generation of DCs

Plastic adherence of peripheral blood allowed the generation of human moDCs, as previously described [8]. These adherent cells were differentiated in RP10 medium. GM-CSF (100 ng/ml; Leukine, Liquid Sargramostim) and IL-4 (20 ng/ml; R&D Systems) were supplemented from the beginning every other day.

### Immunostaining

Fluorescence-labeled, monoclonal antibodies commercially available from BD Biosciences, DakoDiagnostika, Immunotech, R&D Systems and eBioscience were used for staining of generated DCs.

### Determination of cytokine production

Cytokine secretion was analyzed using the eBioscience™ ProcartaPlex Human Th1 /Th2 Zytokin-Panel (11-plex) (Thermofisher) according to the manufacturer’s instructions.

### Mixed lymphocyte reactions

1 × 10^5^ allogeneic peripheral blood mononuclear cells were co-cultured with pretreated and irradiated stimulator moDCs. On day 5, after a 16 h pulse with [^3^H]-thymidine (18.5 kBq/well; GE Healthcare) Tritium-labeled thymidine incorporation was measured.

### Detection of apoptosis

Apoptosis in DCs was detected by live-dead staining using the propidium iodide or 7-aminoactinomycin D–annexin V staining kit from eBioscience.

### Polyacrylamide gel electrophoresis and western blotting

Western blotting was performed as described before [9]. Briefly, whole cell lysates were generated, and protein concentration was measured using a bicinchoninic acid assay (Pierce, Perbio Science, Bonn, Germany). Whole cell lysates (20 µg) were separated on a polyacrylamide gel and transferred onto a nitrocellulose membrane. Monoclonal antibodies by Santa Cruz Biotechnology Inc. (Santa Cruz, USA) were used. An enhanced chemiluminescence kit was used to detect protein bands (GE Healthcare).

### Statistical analysis

All experiments were performed at least 3 times, with representative experiments shown. Statistical significance was calculated with one-way analysis of variance (ANOVA) and Dunnett’s using the Prism 8.4.3 software (Graphpad Software).

## Results

### Idelalisib modulates the differentiation of human monocytes into DCs and decreases LPS-induced maturation of moDCs

**Fig. 1:**
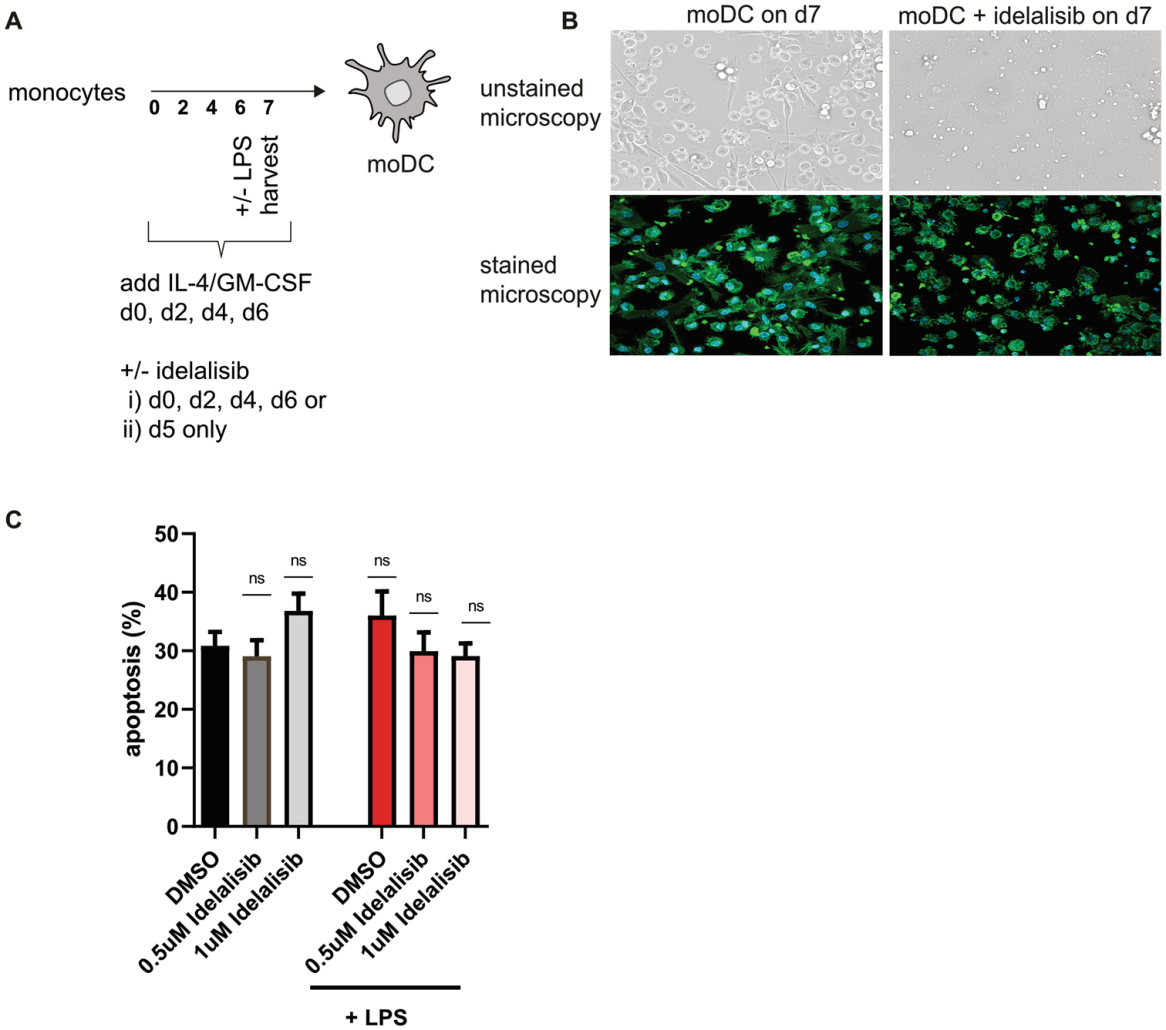
Idelalisib modulates the phenotype of moDCs without affecting cell viability **A** Schematic experimental design for the differentiation of moDCs. Human monocytes cultured under DC-driving conditions were exposed **(i)** every other day to different concentrations of Idelalisib (0.5 µM, 1 µM and 2 µM on day 0, 2, 4 and 6) or DMSO or **(ii)** only on day 5. When indicated, TLR stimulation was applied on day 6. Cells were analyzed on day 7. **B** Representative pictures of unstained and Phalloidin/DAPI-stained moDCs after Idelalisib pre-treatment are shown, **C** Apoptosis rate calculated after PI and Annexin V staining is shown. MoDCs were generated in the presence of Idelalisib. Idelalisib or DMSO was added every other day. (0.5 µM, 1 µM)

To understand dose-dependent effects of the PI3Kδ inhibitor on lineage and activation markers, we differentiated monocytes into moDCs in the presence of GM-CSF and IL-4. Idelalisib applications were performed from day 0, on every other day, and final LPS maturation was induced on day 6 (Fig. 1A). Chosen concentrations of Idelalisib correlate with patient´s sera concentrations.

**Fig. 2:**
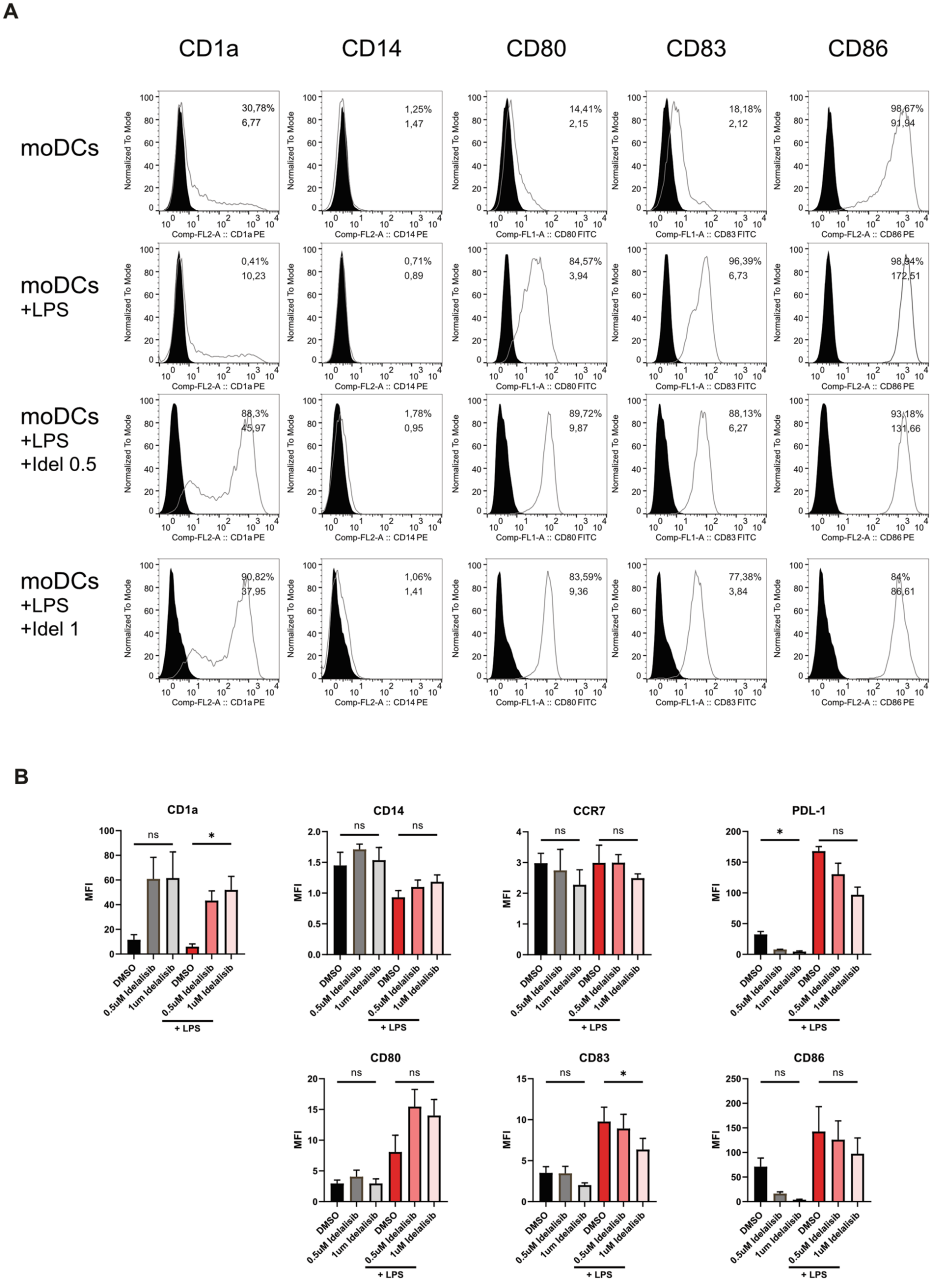
Modulation of co-stimulatory molecules by Idelalisib pre-treatment **A** MoDCs were generated in the presence of IL-4 and GM-CSF. Idelalisib was added every other day followed by subsequent LPS maturation on day 6, when indicated. Cells were harvested on day 7 and analyzed for expression of surface markers via FACS. Plots show mean fluorescent intensity (MFI) from one representative experiment. **B** MFI of surface molecules from three representative experiments were pooled and are shown. The significance was calculated according to the one-way ANOVA Dunnett multiple comparison test and is related to the vehicle control. *P < 0.05

As a first approach, we investigated the effect of Idelalisib on DC morphology and observed impaired formation of dendritic branches and trees. The generated cells resembled monocytes rather than DCs. (Fig. 1B). Next, we analyzed whether Idelalisib induces apoptosis in DCs, but did not detect any significant toxic effects on DCs in the concentrations used in vitro. (Fig. 1C). Expression of the monocyte marker CD14 was not affected by Idelalisib treatment and remained stable, whereas CD1a, a surface protein important for the presentation of lipid and glycolipid antigens, was significantly upregulated by Idelalisib treatment after LPS maturation in a dose-dependent manner. LPS-matured DCs showed significantly reduced CD83 expression when compared to vehicle-exposed monocytes, while other activation markers such as CD86 and CD80, were not affected. PI3Kδ blockade also resulted in downregulation of PD-L1 on immature moDCs, while CCR7 expression was not affected by Idelalisib. However, the effect on PD-L1 expression was not statistically significant after LPS maturation (Fig. 2A and B).

### Allogeneic T cell activation by human moDCs is dampened by Idelalisib treatment

**Fig. 3:**
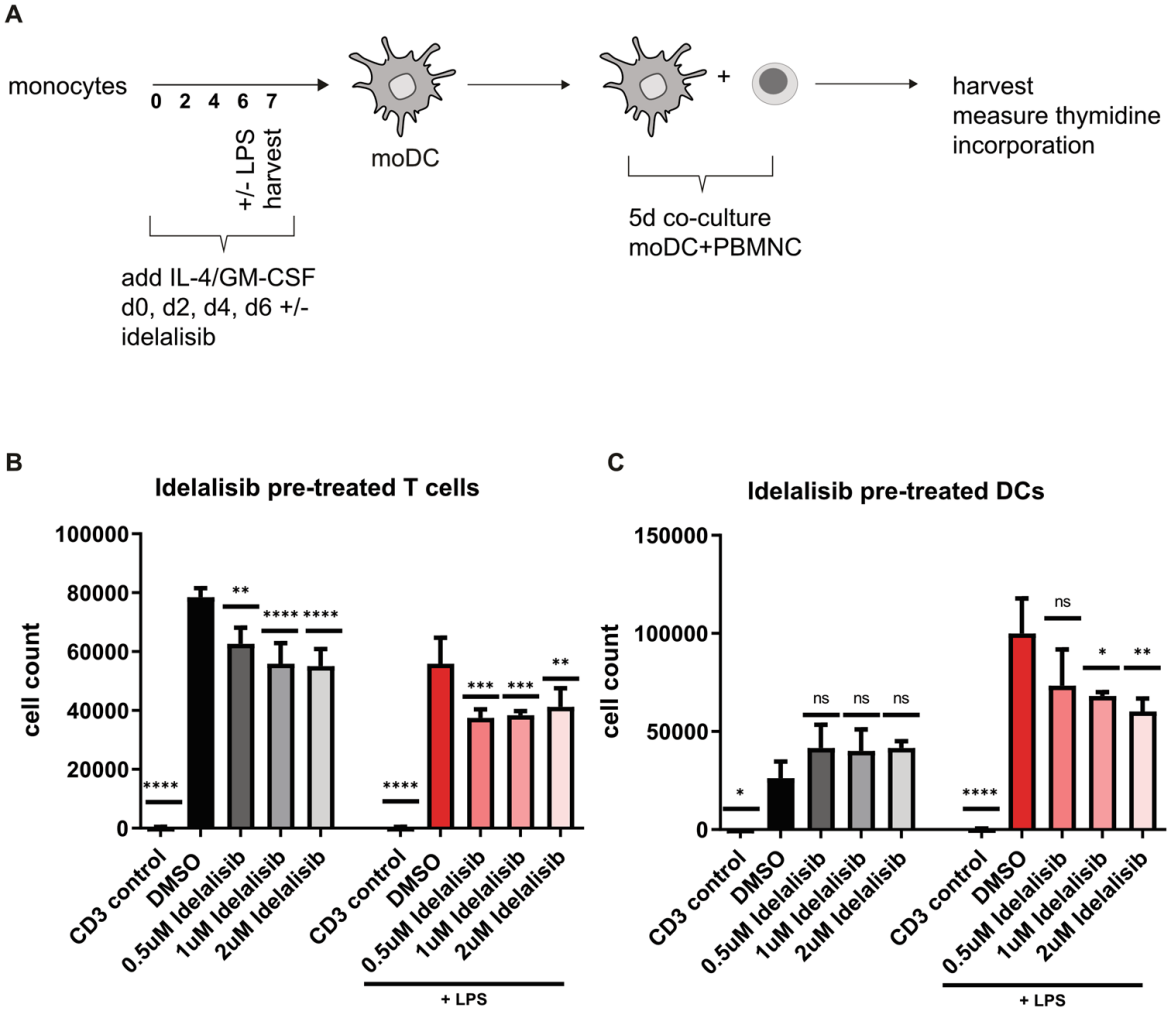
Idelalisib affects proliferation of allogeneic T cells in a DC-dependent manner **A** Schematic experimental design for a mixed lymphocyte reaction. Human monocytes cultured under DC-driving conditions with or without final LPS stimulation were exposed every other day to different concentrations of Idelalisib (0.5 µM, 1 µM and 2 µM on day 0, 2, 4 and 6▪) or DMSO throughout the differentiation period. Afterward, moDCs were co-cultured with allogeneic cells for five days. Thymidine incorporation was measured, and cell numbers were calculated. **B** T cells were co-incubated for 24 h with Idelalisib. (0.5 µM, 1 µM, 2 µM). Washed cells were co-incubated with differentiated moDCs naïve for Idelalisib treatment for 5 days and T cell counts 16 h after a 16 h pulse with [^3^H]-thymidine are shown. Filled black graphs represent negative controls. **C** Monocytes were cultured under DC-driving conditions and treated with Idelalisib (0.5 µM, 1 µM, 2 µM) every second day (day 0, 2, 4, 6), followed by LPS activation on day 6, when indicated. Pretreated moDCs were co-cultured with untreated T cells for an additional five days. T cell counts 16 h after a 16 h pulse with [^3^H]-thymidineare shown. Filled black graphs represent negative controls

We were further interested in how Idelalisib modulates the DC –T cell interaction. First, we were able to show that T cells treated with Idelalisib showed reduced proliferation when co-incubated with allogeneic moDCs. (Fig. 3B) To assess the capacity of moDCs to orchestrate allogeneic T cell proliferation, we co-incubated Idelalisib-pretreated moDCs and untreated T cells. This mixed lymphocyte reaction (MLR) showed impaired induction of allogeneic T cell proliferation by Idelalisib-pretreated moDCs in vitro (Fig. 3C). Thus, Idelalisib affects both T cells and DCs as it reduces their capacity to induce allogeneic T cell responses.

### Idelalisib alters the cytokine profile of LPS-matured DCs

**Fig. 4:**
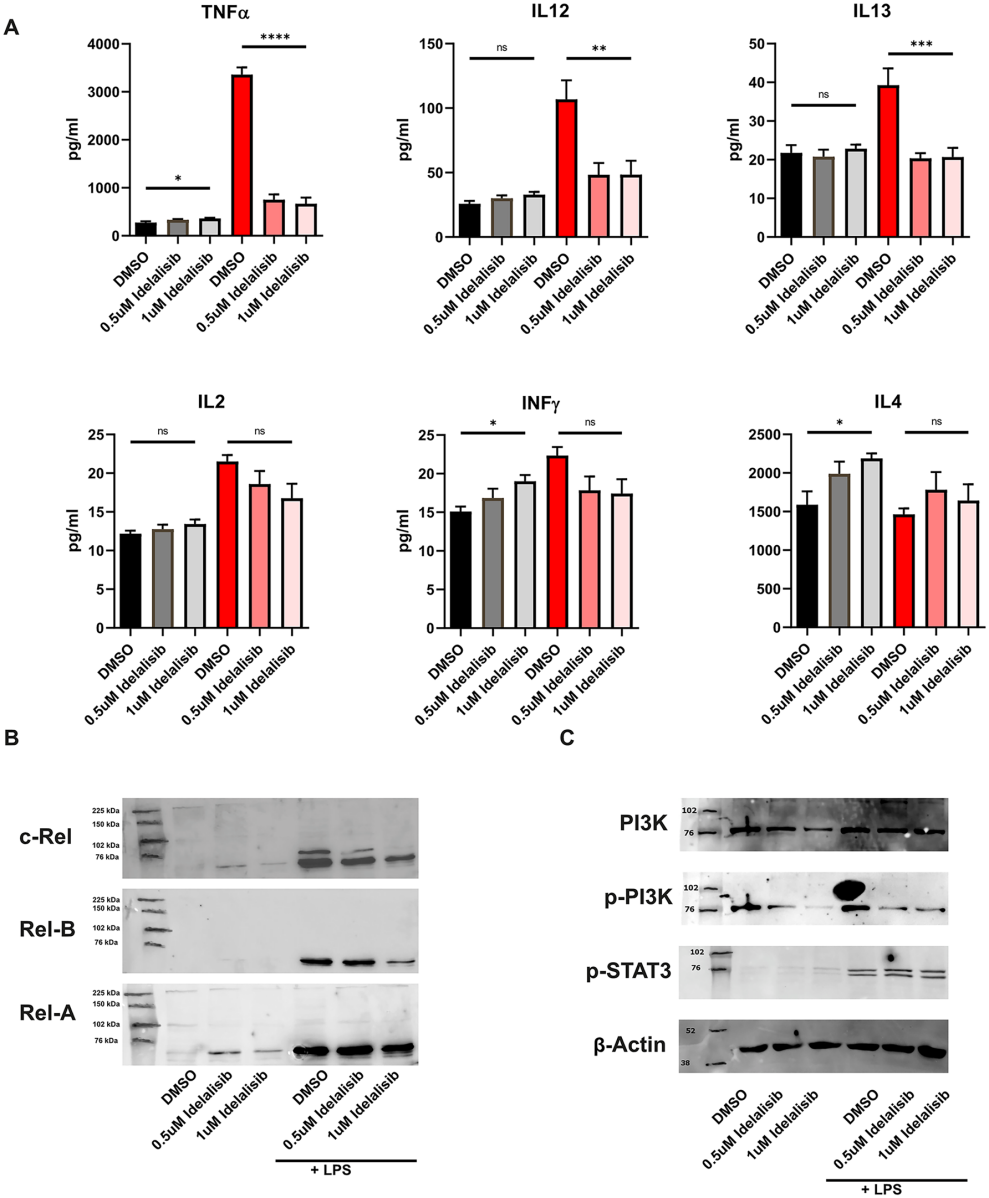
Idealisib alters cytokine production by LPS stimulated moDCs and interferes with the NFκB pathway. **A** MoDCs were generated as described in the presence of Idelalisib followed by subsequent LPS maturation on day 6 when indicated. Supernatants were collected on day 7 and analyzed for cytokine expression. The significance was calculated according to the 1-way ANOVA Dunnett multiple comparison test and is related to the vehicle control. *P < 0.05; **P < 0.01; ***P < 0.001, P < 0.0001. **B** MoDCs generated in the presence of Idelalisib or DMSO were partly stimulated with LPS on day 6. 24 h later, protein was isolated, and lysates were analyzed for Rel-B, c-Rel and Rel-A expression. Representative western blots show upregulation of Rel-a, Rel-B and c-Rel by TLR stimulation. Rel-B and c-Rel expression is dose-dependently inhibited by Idealisib. Rel-A expression is unaffected by Idealisib exposure and serves as a loading control. **C** Protein was harvested from pretreated MoDCs as described above. Immunoprecipitates were analyzed for expression of PI3K, phosphorylated PI3K, phosphorylated STAT3 and β Actin by western blotting. Representative blots are shown. Idelalisib does not affect expression of PI3K and p-STAT3, but phosphorylation of PI3K is inhibited by Idealisib. Equal protein amounts are confirmed by β Actin expression

Besides co-stimulatory signaling and antigen presentation, secretion of cytokines influences the induction of an immune response. Therefore, pro-inflammatory cytokines were analyzed after Idelalisib treatment and LPS stimulation. IL-12 and IL-2 are produced by DCs and are essential for T cell activation. Interferon γ (INF γ), IL-4 and IL-13 induce or maintainT cell polarization. TNFα as a T cell effector molecule was measured. We detected significantly diminished levels of TNFα, IL-13 and IL-12 in Idelalisib-pretreated moDCs while levels of IL-2 and INF γ remained unchanged. In contrast, IL-4 secretion was even upregulated by Idelalisib treatment of immature moDCs, but this effect was abolished in mature moDCs (Fig. 4A).

### Idelalisib inhibits the NFkB pathway downstream of PI3Kδ

Due to significant downregulation of relevant cytokines for T cell proliferation and function, we were interested in the NFκB pathway, which is downstream of PI3K. For this purpose, we analyzed protein expression of Rel-B and c-Rel. LPS-induced upregulation of both proteins in moDCs, which was reduced by Idelalisib pre-treatment in a dose-dependent manner. Rel-A remained unchanged. (Fig. 4B). As expected, phosphorylation of PI3K was inhibited by Idelalisib. Neither endogenous PI3K expression nor expression of p-STAT3 were altered. (Fig. 4C).

## Discussion

In the past decade, the treatment of Non-Hodgkin B cell lymphoma, such as FL and CLL, has evolved due to the discovery of novel drugs. Among small molecules and targeted therapies, Idelalisib, an inhibitor of PI3Kδ, was a promising new candidate.

Nevertheless, pneumonitis, elevation of transaminases, colitis and diarrhea, as well as life-threatening infections, are serious side effects [3, 5] and have limited the treatment. Moreover, it has been shown that T cell-mediated immunity is dampened by Idelalisib treatment [6], and infectious complications are frequent [5]. Therefore, cytomegalovirus (CMV) monitoring as well as pneumocystis jirovecii pneumonia (PJP) prophylaxis are now mandatory. These infectious complications have led to a decreased and very cautious use of Idelalisib, and its replacement, by other new compounds in many cases in clinical routine [5]. However, the exact mechanisms mediating the immunosuppressive potential of Idelalisib have not been elucidated in detail yet.

Our report highlights that not only T cells and malignant B cells are targeted by PI3Kδ inhibition but also DC differentiation and function are deeply modified by Idelalisib exposure in vitro. Differentiation of monocytes into moDCs using GM-CSF and IL-4 was markedly impaired by Idelalisib, which modulated proper DC differentiation and phenotype. For example, expression of CD83, which identifies mature DCs and is the most potent co-stimulatory molecule in the induction of allogeneic T cell proliferation, was impaired upon LPS exposure and Idelalisib treatment [10]. A decrease of CD83 expression due to Idelalisib co-culture may therefore emphasize the immature phenotype of these cells as well as their inability to induce T cell proliferation. As shown previously by a siRNA approach targeting human DCs in vitro [11], the downregulation of CD83 alone is sufficient for impaired T cell responses, even if expression of other co-stimulatory molecules is unaffected. The impaired DC function is further emphasized by diminished IL-12, IL-13 and TNFα secretion after LPS challenge. IL-13 production by DCs is important to maintain cytokine production in T helper 2 (Th2) cells [12]. IL-4, which is unchanged by Idelalisib treatment in mature moDCs, induces Th2 differentiation [13]. IL-13, in contrast, orchestrates survival and functionality of Th2 cells [12, 14]. Th2-type cytokines are increased during CMV infection [15] and proper cytokine production by Th2 cells, which is maintained by IL-13 derived from DCs, is necessary for CMV clearance.

IL-12 is a central cytokine in the induction of T cell responses [16], and adequate release of IL-12 is required to control primary CMV infection in a CD4 + T cell-dependent manner [17]. Aside from lymphopenia, the impaired release of IL-12 and IL-13 by DCs may thus contribute to the increased susceptibility of Idelalisib-treated patients to CMV infection. Additionally, we were able to show that direct inhibition of the NFκB pathway may be one-way how Idelalisib impairs DC function and phenotype. In line with our data, previous studies could underline the importance of the NFκB pathway in DC development and IL-12 production [18].

Another serious complication during Idelalisib treatment is PJP infection. It has been shown previously that TNFα is required for clearance of pneumocystis jirovecii [19] and reduced TNFα levels, as shown here, may be why Idelalisib treatment increases the risk of fatal PJP infection. However, our results are limited because we only investigated the effect of Idelalisib on DC function and not on macrophages, which are a biologically significant source for TNFα.

Last, the inability of Idelalisib pretreated DCs to induce robust immune responses is stressed by the fact that DCs exposed to Idelalisib improperly stimulate allogeneic T cell proliferation, which is another prerequisite to induce a robust immune response after an infectious challenge. DCs activate naïve T cells via 3 signals: Antigen presentation (signal 1), co-stimulation (signal 2) and cytokine production (signal 3) [20]. Although we did not investigate antigen presentation, we could unveil modulation of co-stimulation and cytokine production by Idelalisib, which is further supported by our results regarding the induction of allogeneic T cell proliferation.

Our results may help to understand the restricted use of Idelalisib due to fatal infectious complications. Idelalisib modulates the expression of at least one co-stimulatory molecule and, more importantly, diminishes cytokine production by DCs in vitro. These cytokines are essential for proper T cell differentiation and polarization. Clinical infection with CMV and PCP may therefore not only be caused by defective T cell responses, but also by insufficient T cell polarization due to diminished cytokine release. Although our data were generated in vitro, clinical observations implicate relevance for our findings in vivo.

In contrast, suppression of T cells and DCs may open new therapeutic venues for PI3Kδ inhibition. For example, in myeloproliferative neoplasms, which are driven by chronic inflammation [21], a clinical trial combining PI3Kδ inhibition and standard treatment with the JAK inhibitor Ruxolitinib showed promising interim results in patients with myelofibrosis (ClinicalTrials.gov Identifier: NCT02718300). These promising results are supposed to be caused by direct effects of PI3K inhibition on malignant cells [22], but additional effects as shown in this work have to be investigated as well.

## Data Availability

The datasets generated during and analyzed during the current study are available from the corresponding author on reasonable request.
